# Advance directives and end-of-life decision-making in the ICU: results from an observational study

**DOI:** 10.1186/cc11695

**Published:** 2012-11-14

**Authors:** CS Hartog, I Peschel, D Schwarzkopf, B Kabisch, A Günther, R Pfeifer, H Skupin, K Reinhart, N Riedemann

**Affiliations:** 1 University Hospital Jena, Friedrich-Schiller University, Jena, Germany; 2 Center for Sepsis Control and Care, Integrated Research and Treatment Center, University Hospital Jena, Jena, Germany

## Background

Advance directives (AD) are intended to ensure patient autonomy in the event of loss of decision-making capacity. According to German law, ADs are now legally binding. It is unclear whether they are helpful in the ICU [1]. The aim of this study is to compare ICU treatment with patients' written wishes.

## Methods

The setting was surgical, neurological and cardiological ICUs of one German university hospital with a total of 72 beds. All patients with AD who died on the ICU between December 2010 and December 2011 were included. ADs were collected from patients' files, and their exact specifications, wishes and preferences were extracted and tabulated. Patient demographics and medical treatment was extracted from an Electronic Patient Data Management System. The time period was 48 hours preceding death. AD were defined as *applicable *if they described the potentially applicable clinical situation as 'final stage of a fatal disease' or 'inevitable immediate dying process'. In contrast, AD were defined as *not applicable *if they described other situations, including 'mental deterioration', 'breakdown of vital organs' or '(in)direct brain damage'.

## Results

Sixty-four ICU patients were included. AD were written a median 109 weeks (IQR 26.95, 261.11 weeks) before the ICU stay. Forty-four AD were applicable according to the definition. Of 44 patients with applicable ADs, 50% were surgical patients, 41% patients had severe sepsis. Within 48 hours prior to death, 27% received a DNR/I (do-not-resuscitate/intubate) order or DND (do-not-dialyze) order. Eighteen per cent received a decision to withhold, 43% a decision to withdraw therapy. Twenty-two patients (50%) refused intensive care, 98% (43/44) specifically refused life-sustaining measures and, in addition, many specifically named measures to be avoided. Patients who refused life-sustaining measures received the following treatments: within 48 hours prior to death, 12% received CPR and 23% underwent surgery. Within 24 hours, 77% were mechanically ventilated and on vasopressors. At the time of death, 72% were intubated, 74% with FiO_2 _>21%, 30% on vasopressors. Of these 43 patients, 21 were surgical and 22 nonsurgical patients. Fourteen out of 43 applicable AD were pre-printed forms, 29 were written individually. No trend was noticeable regarding compliance with single patients' wishes in these subgroups.

## Conclusion

AD do not seem to direct end-of-life care in the ICU. Our findings strengthen concerns that AD in their current form may not be suitable for this patient population. Moreover, inability to comply with patients' wishes may add to the emotional burden of ICU staff.
